# Self-concept explains gender differences in mental rotation performance after stereotype activation

**DOI:** 10.3389/fpsyg.2023.1168267

**Published:** 2023-05-15

**Authors:** Martina Rahe, Linda Schürmann, Petra Jansen

**Affiliations:** ^1^University of Koblenz, Universitaetsstrasse, Koblenz, Germany; ^2^University of Regensburg, Universitaetsstrasse, Regensburg, Germany

**Keywords:** mental rotation, stereotype activation, self-concept, perceived abilities, gender differences

## Abstract

**Introduction:**

Mental rotation is an ability that usually displays robust and large gender differences in favor of boys and men. When participants are told that males outperform females, it can result in worse performance for females (stereotype threat effect) and better performance for males (stereotype lift effect). Because various studies show different results for the effect of stereotype activation, the present study aimed to investigate whether adolescents’ self-concept would moderate stereotype activation effects.

**Methods:**

127 adolescents (61 male, 66 female) between 10 and 18  years (*M* = 13.54, *SD* = 1.99) solved the mental rotation test and filled out a questionnaire about their perceived performance, stereotype beliefs, and self-concept.

**Results:**

Results showed that self-concept and stereotype activation affected male and female adolescents’ actual and perceived performance differently. For males, a better self-concept was associated with better performance in adolescents with stereotype activation and with lower performance in those without the activation. This interaction was only marginally significant. For females, an increasing self-concept was associated with worse performance in adolescents with stereotype activation and with better performance in those without activation. Furthermore, a better self-concept was associated with higher perceived performance in male adolescents with stereotype activation and with lower perceived performance without the activation.

**Discussion:**

Reasons for these results could be the (in)congruence between males’ own perception of their abilities (self-concept) and the instruction of the activation.

## 1. Introduction

Mental rotation is a spatial ability defined as rotating two or three-dimensional objects in imagination (Shepard and Metzler, 1971; Voyer et al., 1995). Studies show the importance of spatial abilities, e.g., for adolescents’ choice of advanced education in STEM subjects (Wai et al., 2009). Small male advantages in mental rotation performance appear in children under 13 (mean weighted *d* = 0.33). They increase with age to large gender differences (mean weighted *d* = 0.66) in adults (Voyer et al., 1995). In a meta-analysis, Lauer et al. (2019) took a closer look at children and adolescents up to 17 years and found small gender differences in childhood (mean weighted *g* = 0.20 at 6 years) that increased to a medium effect size during adolescence (mean weighted *g* = 0.50 at 14 years).

In a study with young adolescents (*M* = 12.5 years) and young adults (*M* = 22.2 years), age and mental rotation performance correlated significantly for males (*r* = 0.43), but not for females (*r* = 0.05) (Rahe and Quaiser-Pohl, 2023). This illustrates increasing gender differences with age because of a lack of improvement of abilities in females during adolescence. Furthermore, participants were asked about their perceived performance in the mental rotation task. Correlations of perceived performance and age were significantly negative for females (*r* = −0.35) and non-significant for males (*r* = 0.19). This shows that young women may perceive their own performance as worse than girls who are 10 years younger. Stereotypes can influence spatial performance (Hausmann et al., 2009; Rahe et al., 2021; Rahe and Jansen, 2022), and stereotype influence might be moderated by self-concept (Rossi et al., 2022). Moreover, during adolescence, gender differences in actual and perceived performance seem to increase. Therefore, we took a closer look at the effects of stereotype activation, self-concept, and gender on actual and perceived mental rotation performance at this age.

### 1.1. Stereotype activation effects on mental rotation performance

When people are asked which gender performs better in various cognitive tasks, the percentage of correct answers in mental rotation tasks is rated as male-stereotyped (Halpern et al., 2011). These beliefs are dependent on people’s preoccupation. Science students are more likely than art students to believe that someone good in mental rotation was likely to be male (Hausmann, 2014). Moreover, male participants rate their performance in mental rotation tests more positively than women (Cooke-Simpson and Voyer, 2007; Estes and Felker, 2012; Rahe and Jansen, 2022). Again, science students’ self-ratings were higher than art students’ (Hausmann, 2014). Hence, most people are aware that men outperform women in such abilities. This conviction is already evident in children as young as elementary school age (Neuburger et al., 2015; Moè, 2018a). It can result in stereotype threat effects.

Stereotype threat is the “risk of confirming, as self-characteristic, a negative stereotype about one’s group” (Steele and Aronson, 1995, p. 797). It can affect performance in cognitive tasks negatively. In contrast, when people believe that an outgroup underperforms in a cognitive task, they may experience a stereotype lift (Walton and Cohen, 2003) and show better performance. Stereotype threat or lift effects regarding gender differences in spatial abilities can be activated directly by telling participants that one gender outperforms the other (Heil et al., 2012). Alternatively, these effects can be induced indirectly by using gender-stereotyped material as rotational objects (Ruthsatz et al., 2017; Rahe et al., 2021; Rahe and Jansen, 2022) or by asking participants to decide which gender would more probably outperform the other gender in certain activities (Hausmann et al., 2009).

When gender stereotypes are experimentally activated in a spatial task, many studies show that participants’ performances increase under a stereotype lift condition and decrease in a threatening condition (Hausmann et al., 2009; Rahe and Jansen, 2022). This effect also appeared in children (Neuburger et al., 2015; Ruthsatz et al., 2017). However, other studies showed no effects of stereotype threat activation on mental rotation performance in adults (Bauer et al., 2021). Reasons for these various results could be different sample sizes, age ranges of the participants, and different stereotype activations. For example, both Neuburger et al. (2015, 216 children in 4th grade) and Ruthsatz et al. (2017, 321 children in 2nd and 4th grade) tested children. In both studies, small effect sizes of the interaction of stereotype activation and gender were found for fourth graders, *η*^2^ = 0.04, but Ruthsatz et al. (2017) found no effect for second graders. This gives a hint that gender stereotypes cannot be successfully activated in children as young as seven or 8 years.

Bauer et al. (2021, 107 participants) and Rahe and Jansen (2022, 117 participants) tested young adults. Bauer et al. (2021) used a chronometric task, where gender differences are usually smaller (Voyer et al., 1995) and could be the reason why gender stereotype activation effects could not be detected. Rahe and Jansen (2022) used a within-subjects design with male and female-stereotyped objects as rotation material and found a significant interaction of gender and material (*η*^2^ = 0.06), indicating that men solved male-stereotyped objects better and women were more successful solving female-stereotyped objects. Hence, a stereotype activation induced by the rotational material seems to be successful in fourth-grade children (Neuburger et al., 2015; Ruthsatz et al., 2017) and adults (Rahe and Jansen, 2022). This is in line with findings of a meta-analysis on stereotype threat and lift effects on math and spatial performance displaying significant stereotype lift effects on females for spatial tasks (weighted estimator of effect size = −0.39), while stereotype threat effects (weighted estimator of effect size = 0.12) were not (Doyle and Voyer, 2016). Another meta-analysis on the effects of stereotype threat activation on girls’ performances in math, science, and spatial skills showed significant negative effects of the activation (weighted estimator of effect size = −0.22, Flore and Wicherts, 2015). Accordingly, stereotype activation is a crucial aspect to consider when researching spatial performance in adolescents.

### 1.2. Stereotype activation effects on adolescent participants

Of the four studies with adolescent participants, Dunst et al. (2013, 63 adolescents between 15 and 18 years) and Rahe and Jansen (2023, 402 adolescents between 5th and 12th grade) told one group that men outperformed women in mental rotation while the second half got no information about gender differences. Both studies found no significant effect of the stereotype threat activation, although the interaction of gender and stereotype activation showed a small, insignificant effect in the study by Dunst, *η*^2^ = 0.04, compared to a lack of effect in the study by Rahe and Jansen, *η*^2^ = 0.005. In the study by Dunst (2013), participants were excluded when they disagreed with one of the statements “I am good at math” or “It is important to me that I am good at math.” Hence, this sample seems to have had better perceived mathematical abilities and attributed greater importance to mathematics than the participants in the study by Rahe and Jansen (2023), who were more heterogeneous in their mathematical abilities. This could have an influence on the possibility of successfully activating gender stereotypes.

Moè and Pazzaglia (2006, 197 adolescents between 16 and 18 years) tested an additional third group. The participants got the information that women outperformed men and were asked about their beliefs about whether men or women would perform better in spatial tasks. Moreover, Moè and Pazzaglia (2006) tested participants’ mental rotation performance twice, before and after the instruction, and ran the analyses for males and females separately in a within-subjects design. The performance of female participants increased after the instruction that women were better, especially when participants thought that no gender differences existed prior to the experiment. Females’ performance decreased when they were told that men were better, especially when their prior beliefs were that men would be better. For male adolescents, the performance increased after the instruction that men were better, especially when their prior beliefs were that men were better in mental rotation. Males’ performance decreased after the instruction that females were better, especially in participants who thought that there were no gender differences prior to the experiment. Effect sizes in this study were large for male and female adolescents, which underlines the relevance of Moè and Pazzaglia’s study. Moè and Pazzaglia (2006) included prior beliefs and found distinct effects for adolescents who thought that men would outperform women and those who thought that no differences existed.

Rahe and Quaiser-Pohl (2019) used cube and pellet figures to activate gender stereotypes and found no interaction between gender and stimulus material, *η*^2^ = 0.001. Compared to the studies of Neuburger et al. (2015) and Ruthsatz et al. (2017), who used gender-stereotyped real objects, e.g., cars and dolls, the use of pellet figures instead of cube figures could not diminish gender differences (Rahe and Quaiser-Pohl, 2019).

To summarize, studies and meta-analyses show different effects of stereotype threat activations on the performance of male stereotyped abilities. The question arises if stereotype threat effects degrade performances only in some participants or only under certain conditions. One approach would be to take a closer look at adolescents’ self-concept as a possible moderator of stereotype activation effects on male and female participants’ mental rotation performance.

### 1.3. Moderator effects of stereotype activation

Guizzo et al. (2019) told their participants that men outperform women in mental rotation tasks (stereotypical) or that there are no gender differences (stereotype-nullifying) and assessed participants’ implicit beliefs and their mental rotation ability. Results showed a significant three-way interaction of gender, condition, and implicit beliefs, *b* = 4.01, SE *b* = 1.52. The stronger the associations of space (or spatial abilities) and men were, the worse the men in the stereotype-nullifying condition performed. The authors suggest that the incongruence of men’s associations between space and men and the explicit information of the experimenter that both genders perform equally well diminished men’s performance.

Activating stereotype threat in science and art students, Hausmann (2014) found a three-way interaction of academic discipline, gender, and stereotype threat on participants’ mental rotation performance, *η*^2^ = 0.07. A stereotype threat affected female art students negatively, whereas female science students performed even better in the stereotyped than in the non-stereotyped condition (Hausmann, 2014). Hausmann explained the enhanced performance of women in science with a counter-stereotypic group identity. He suggests that these women were more self-confident and/or less anxious and, hence, were less susceptible to gender stereotype threat.

Delgado and Prieto (2008) found that stereotype threat activation did not have different effects on male and female adolescents’ math performance (no significant interaction of stereotype condition and gender). However, if math anxiety was considered, results showed that females’ performance under threat compared to the non-threat condition decreased in participants with high anxiety and increased in those with low anxiety (Delgado and Prieto, 2008). This can be explained by the threat-related attentional bias (Bar-Haim et al., 2007) that seems to affect only anxious people (Delgado and Prieto, 2008).

Wang et al. (2018) tested academically low-achieving students and activated a stereotype threat by presenting a statement for 1,000 ms about low achievement before each mental rotation item. Results showed that performances diminished particularly in students with high self-esteem. The authors suggest that adolescents with low self-esteem are already under threat. Gerstenberg et al. (2012) assessed females’ implicit and explicit math self-concepts and found diminished performance only in females with low implicit and high explicit self-concepts. Other studies found that stereotype threat effects were reduced when the girls’ mothers had no gender stereotype beliefs (Tomasetto et al., 2011) and when the task was perceived as malleable (Good et al., 2003). The summarized studies showed that stereotype threat and lift effects in gender stereotypical tasks were dependent on people’s self-esteem, self-confidence, self-concept, anxiety, and identity as either masculine or feminine.

Also considering gender, Rossi et al. (2022) conducted a study with young adults who were asked about their mathematics self-concept, gender stereotypes of mathematics, and math anxiety and who solved a math test. Results showed that gender stereotypes affected the self-concept differently in men and women and that the self-concept was associated with test performance. This gives a first hint that gender stereotypes could have different effects on test performance in men and women in relation to their self-concept. Our study extends this by using an experimental approach. It aims to investigate these different effects of self-concept on gender differences in mental rotation after stereotype activation to help girls and young women live up to their potential.

### 1.4. The goal of the study

The present study analyzed the effects of stereotype activation on male and female adolescents under consideration of the self-concept. Thereby, it closes the research gap regarding possible interactions between self-concept, stereotype activation, gender, and mental rotation performance. Moreover, it helps with the still inconclusive understanding of why gender differences are found in mental rotation and, consequentially, enables both theoretical and practical implications for mental rotation as an aspect of children’s and adults’ spatial cognition abilities and performance. Adolescents were analyzed as participants because gender differences increase during adolescence (Voyer et al., 1995; Lauer et al., 2019). It is necessary to analyze whether the effects of stereotype activation depend on adolescents’ gender (Moè and Pazzaglia, 2006) and self-concept.

The following hypotheses were investigated: we assumed that stereotype activation effects on adolescents’ mental rotation performance are dependent on gender and self-concept (H1). On the one hand, female adolescents with a lower self-concept should be more susceptible to stereotype threat activation than those with a higher self-concept. On the other hand, the stereotype lift activation effect for male adolescents should be more pronounced in males with a higher self-concept and could even be reversed in males with a low self-concept. The latter could be anxious knowing that males outperform females, but perceiving themselves as not good enough to live up to this stereotype. Furthermore, similar interaction effects are assumed for adolescents’ perceived mental rotation performance (H2).

## 2. Methods

### 2.1. Participants

Participants were 131 adolescents from grades 5 to 11. Two of them did not indicate their age and gender, and two did not answer the SESSKO questionnaire. Hence, 127 adolescents (61 male and 66 female adolescents) between 10 and 18 years (*M* = 13.54, SD = 1.99) remained in the final sample. 15 classes of various grades were tested, and in 7 classes, the stereotype activation was used. No age differences appeared between activated and non-activated participants, *t*(125) = −0.983, *p* = 0.328. The distribution of gender and condition (with/without activation) was equal, *Chi*^2^(1) = 0.376.

A G*Power (Faul et al., 2007) analysis for the regression analyses with a medium effect size *f*^2^ = 0.15 (*α* = 0.05, 1 – *β* = 0.95, three predictors) revealed a total sample size of 119 participants. To our best knowledge, no other study analyzed the effects of gender, self-concept, and stereotype activation on adolescents’ mental rotation performance. Hence, we had no hint of what effect we could expect. Moè and Pazzaglia (2006) found large effects of stereotype activation for girls and boys separately, in different directions (see introduction). Therefore, we assumed a medium effect size for the interaction with self-concept.

### 2.2. Material

#### 2.2.1. Mental rotation test

The mental rotation test consisted of 12 items (re-drawn version according to Vandenberg and Kuse, 1978; Peters et al., 1995). For each item, one main cube figure on the left side had to be compared to four comparison figures on the right side. Two of the comparison figures were identical but rotated versions of the main figure and had to be crossed out. The remaining two figures (distractors) were mirrored versions of the main figure. Objects were rotated by an angle of 45°, 90°, 135°, or 180° to the right or the left. According to the version of Vandenberg and Kuse (1978), all objects consisted of 10 cubes. One point was given only if both identical figures were crossed out. A sum score of all 12 items was calculated. Internal consistency of the scale was acceptable/good (Cronbach’s alpha = 0.802, McDonald’s omega = 0.790).

#### 2.2.2. Self-concept

To assess adolescents‘self-concept, three subscales of the SESSKO were used (*Skalen zur Erfassung des schulischen Selbstkonzepts*, Schöne et al., 2002). The questionnaire has 16 items, which had to be answered on a 5 point rating scale. The questionnaire consisted of questions regarding school (“I think that I am talented for school”), other students (“I can learn better than my classmates”), and the general self-concept (“I am intelligent”). A mean score was calculated. Internal consistency of the scale was excellent (Cronbach’s alpha = 0.952, McDonald’s omega = 0.952).

#### 2.2.3. Gender stereotype beliefs

To assess adolescents’ gender stereotype beliefs, a questionnaire according to Moè and Pazzaglia (2006) with 11 activities was used (Rahe and Quaiser-Pohl, 2019). Five of these activities were male-stereotyped (e.g., mentally rotating objects in one’s mind) and five activities were female-stereotyped (e.g., understanding emotions). One activity was not gender-stereotyped (solving a crossword puzzle). Participants were asked whether males or females were better in each activity on a 7 point rating scale ranging from −3 = *males are better* to 3 = *females are better*. A neutral point (0) indicated that male and female adolescents were equally good at an activity.

A *t*-test revealed that all male stereotyped activities deviated significantly from the neutral point towards the negative scale (all *p*s < 0.001). All female activities except one (“to learn something new”) differed significantly from the neutral point toward the positive scales (all *p*s < 0.001). The neutral activity did not differ significantly from the neutral point (*p* = 0.603). A mean score could not be calculated for male and female stereotyped activities because of low internal consistencies. Therefore, only the variable “mentally rotating objects in one’s mind” was used as adolescents’ belief of a male stereotyped ability. The variable was inverted so that higher values stand for firmer stereotype beliefs.

#### 2.2.4. Perceived ability in masculine and feminine activities

For the perceived ability in masculine and feminine activities, the same 11 activities were presented in a questionnaire (Rahe and Quaiser-Pohl, 2019). Adolescents were asked to rate their ability in each activity on a 6 point rating scale ranging from 1 = *very good* to 6 = *very bad*. This answering scale corresponds to the grading system in Germany. Internal consistency of the scale was below acceptable values (Cronbach’s alpha = 0.691, McDonald’s omega = 0.693). Therefore, only the variable “mentally rotating objects in one’s mind” was used as adolescents’ perceived ability of a male stereotyped activity. The variable was inverted so that higher values stand for higher perceived abilities.

### 2.3. Procedure

Adolescents were tested in mixed-gender groups in their classrooms (Moè, 2018b). To half of the classes (70 participants, 31 male, and 39 female), the experimenter said that the study was conducted to learn more about why males were better in mental rotation than females (stereotype activation). The other 57 adolescents (30 males and 27 females) got no stereotype activation. A Chi^2^-test revealed no deviation from an equal distribution between gender and stereotype activation conditions (*p* = 0.376). First, two practice items were discussed in class, and then the participants solved the mental rotation test. For the 12 test items, adolescents had a time limit of 3 min (Peters et al., 1995).

Afterward, participants filled out the questionnaire answering the questions about their self-concept, gendered stereotype beliefs, and perceived ability of masculine and feminine activities and indicated their age and gender. One parent of each adolescent gave their written informed consent. The study was approved by the Supervisory and Service Directorate (Aufsichts- und Dienstleistungsdirektion, ADD) and conducted according to the ethical guidelines of the Helsinki declaration. Ethical approval for this study was not required in accordance with the conditions outlined by the German Research Foundation, where research that carries no additional risk beyond daily activities does not require Research Ethics Board Approval.

### 2.4. Statistical analyses

First, we calculated a manipulation check to test whether the stereotype activation group had stronger beliefs that mental rotation (item: mentally rotating objects in one’s mind) was more male stereotyped than adolescents in the condition without activation had. According to Lovakov and Agadullina (2021), *d* values of 0.15, 0.36, and 0.65 were considered small, medium, and large. Second, correlations between the study variables were calculated separately for male and female adolescents and for both conditions. This was done to illustrate the directions and effect sizes of correlations for possible interactions between the study variables. According to Lovakov and Agadullina (2021), correlations of 0.12, 0.24, and 0.41 were considered small, medium, and large. Third, main and interaction effects of gender and stereotype activation on participants’ self-concept were analyzed.

To test both hypotheses, two regression analyses (Process Macro Model 3, Hayes, 2018) with the criteria of actual and perceived mental rotation performance were conducted. Predictors were participants’ gender, self-concept, and the stereotype activation condition. According to Cohen (1988) and Field (2013), *R*^2^ was considered small (0.02), medium (0.13), and large (0.26). Adding age as a covariate into both regression models did not change the significance of any result. A reason could be that age did not significantly correlate with self-concept, perceived mental rotation performance, or stereotype belief of mental rotation (all *p*s > 0.15).

## 3. Results

First, the manipulation check revealed that adolescents in the stereotype activation condition (*M* = 0.94, SD = 1.10) had stronger beliefs that mental rotation was male stereotyped, *t*(122) = 2.485, *p* = 0.007, *d* = 0.450, than those in the condition without activation (*M* = 0.43, SD = 1.21). On a closer look, this holds true only for male, *t*(57) = −2.316, *p* = 0.012, *d* = 0.604, but not for female, *t*(63) = −1.274, *p* = 0.104, *d* = 0.323, adolescents.

Second, correlations between the study variables were calculated for male and female adolescents and for both stereotype activation conditions separately (Table 1). For male and female participants in both conditions, actual mental rotation performance correlated with the perceived mental rotation performance. In male adolescents, mental rotation performance correlated with self-concept and stereotype beliefs of mental rotation only in activated participants. The same was found for the correlations between self-concept and perceived performance and stereotype beliefs. For female adolescents, correlations between mental rotation performance and self-concept were medium and non-significant but in opposite directions. Self-concept and perceived mental rotation performance were correlated only in female participants who were not stereotype activated. Descriptive statistics of the study variables can be found in Supplementary Table A.

**Table 1:** Correlations between the study variable.

		1	2	3	4
1	MR		0.435* (−0.129)	0.600** (0.457*)	0.415* (0.178)
2	Self-concept	−0.237 (0.314)		0.621** (−0.119)	0.701** (0.261)
3	Perceived MR performance	0.484** (0.488*)	−0.087 (0.390*)		0.498** (0.373)
4	Stereotype belief of MR	0.050 (−0.018)	0.031 (−0.311)	−0.298 (−0.352)	

Correlations above the diagonal are for male, below the diagonal for female participants. Correlations without the stereotype activation are in brackets, with activation without brackets. MR, mental rotation; *p < 0.05; **p < 0.01.

Third, gender, stereotype activation, and the interaction of gender and stereotype activation had no significant effect on participants’ self-concept (all *p*s > 0.3).

**Figure 1:**
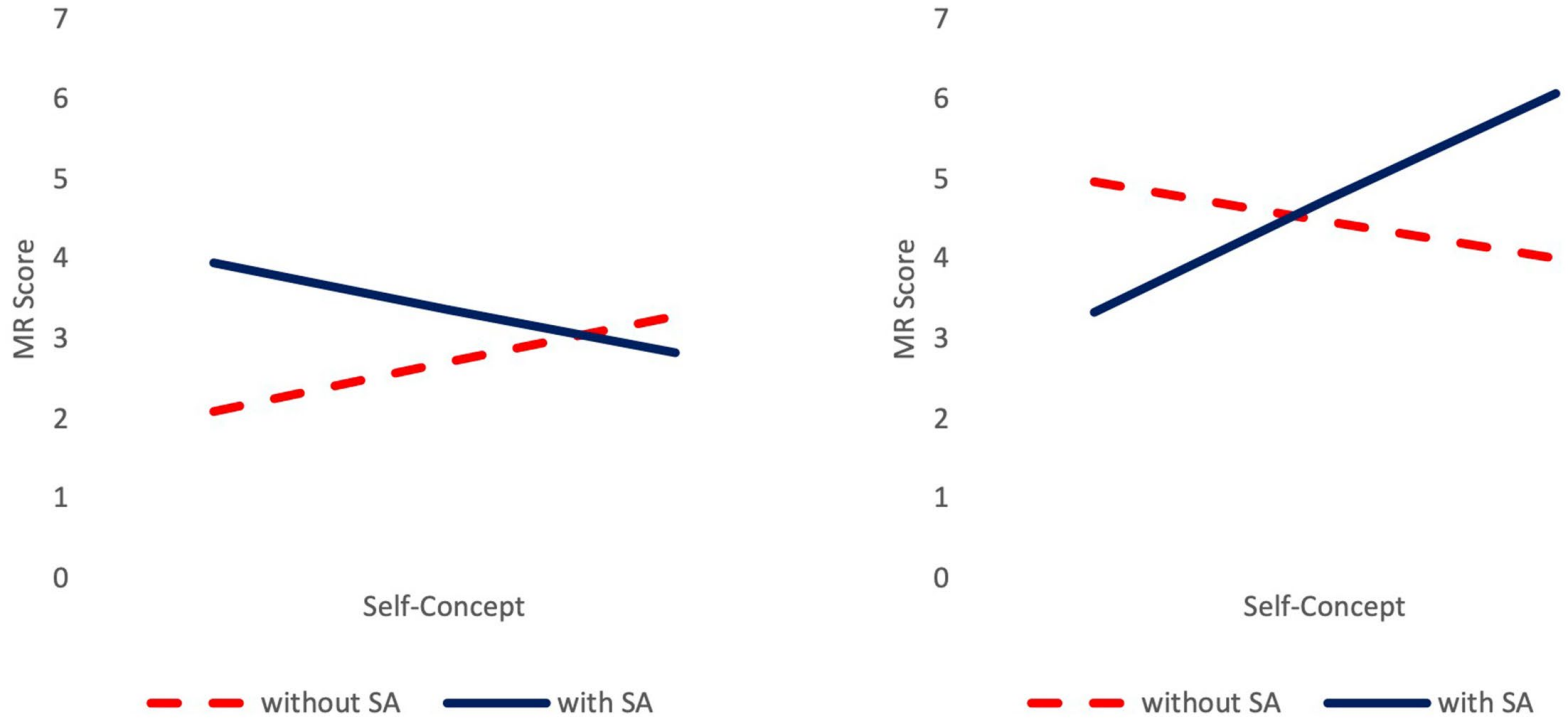
Mental rotation performance as a function of gender, stereotype activation, and self-concept. MR, mental rotation; SA, stereotype activation. Results for female (left) and male (right) adolescents.

For hypothesis 1, a regression analysis was performed to analyze possible predictors of adolescents’ mental rotation performance (Process macro Model 3 by Hayes, 2018). Overall, the model was significant, *F*(7, 119) = 3.840, *p* < 0.001, *R*^2^ = 0.184 (Table 2) with a moderate explanation of the variance of mental rotation performance. The three-way interaction of gender × stereotype activation × self-concept was significant. Figure 1 shows that for female adolescents, an increasing self-concept was associated with better performance in participants without a stereotype activation and with lower performance in those with the activation. A post-hoc regression analysis was performed to analyze a possible interaction of self-concept and stereotype activation on female adolescents’ mental rotation performance (Process macro Model 1 by Hayes, 2018). The interaction of stereotype activation and self-concept was significant (*b* = −1.807, SE = 0.819, *p* = 0.031). For male participants, an increasing self-concept was associated with better performance in adolescents with stereotype activation and with lower performance in those without the activation. A post-hoc regression analysis (Process macro Model 1 by Hayes, 2018) revealed a marginally significant interaction of stereotype activation and self-concept (*b* = 2.884, SE = 1.454, *p* = 0.052).

**Table 2:** Mental rotation score predicted by gender (0 = male, 1 = female), stereotype activation (0 = without activation, 1 = with activation), and self-concept.

	*b*	SE	*t*	*p*	LLCI	ULCI
Constant	7.051	3.756	1.877	0.062	−0.386	14.488
Self-concept	−0.747	1.050	−0.712	0.477	−2.827	1.332
Stereotype activation	−9.652	4.334	−2.226	0.027	−18.234	−1.069
Self-concept × SA	2.884	1.217	2.368	0.019	0.472	5.295
Gender	−7.540	4.512	−1.671	0.097	−16.475	1.394
Self-concept × Gender	1.677	1.283	1.306	0.193	−0.864	4.220
SA × Gender	16.537	5.643	2.930	0.004	5.363	27.711
Self-concept × Gender × SA	−4.691	1.609	−2.915	0.004	−7.877	−1.504

SA, stereotype activation; LLCI, lower-level confidence interval; ULCI, upper-level confidence interval. Confidence intervals are 95%.

**Figure 2:**
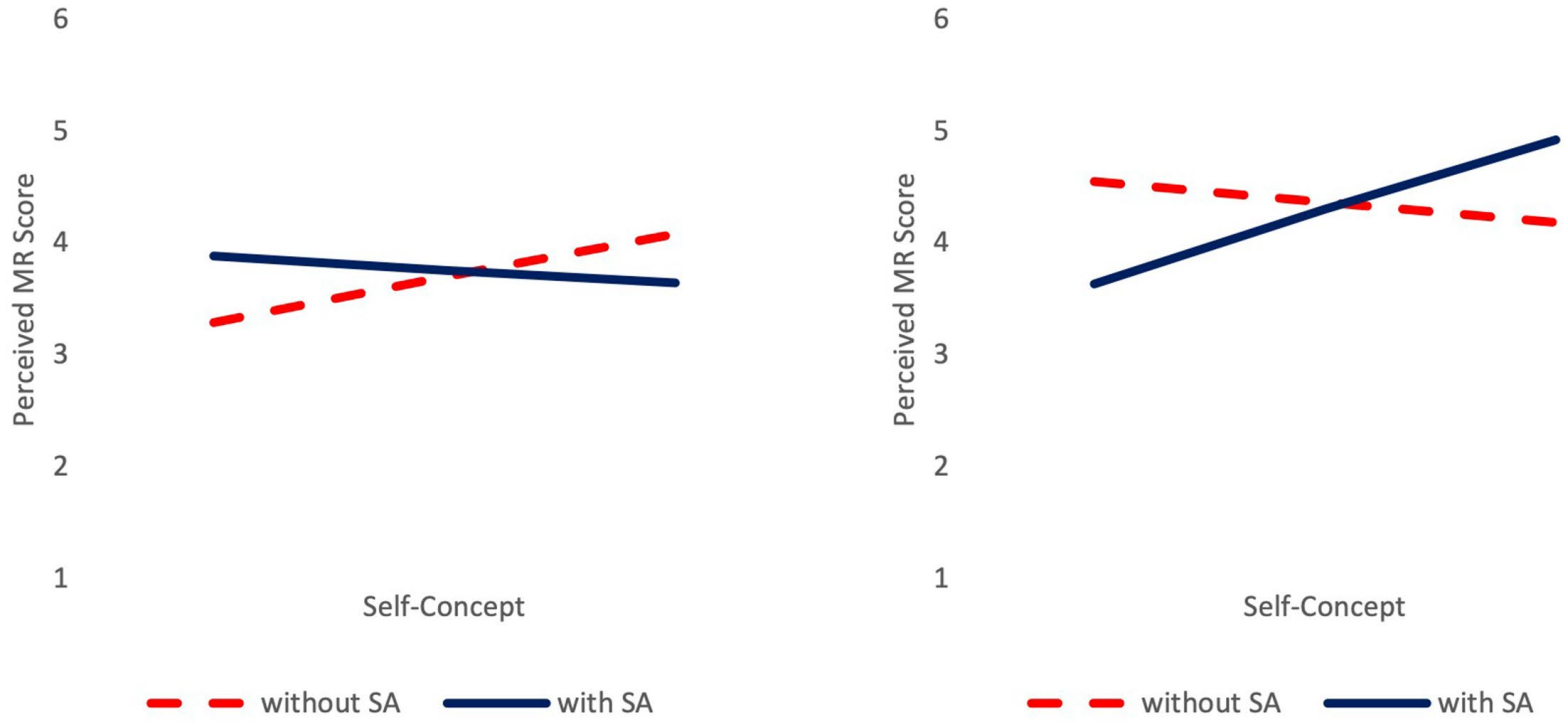
Perceived mental rotation performance as a function of gender, stereotype activation, and self-concept. MR, mental rotation; SA, stereotype activation. Results for female (left) and male (right) adolescents.

A second regression analysis was performed to analyze possible predictors of adolescents’ perceived mental rotation performance (Process macro Model 3 by Hayes, 2018). Overall, the model was significant, *F*(7, 119) = 4.061, *p* < 0.001, *R*^2^ = 0.195 (Table 3) with a moderate explanation of the variance of perceived mental rotation performance. The three-way interaction of gender × stereotype activation × self-concept was significant. Figure 2 shows that for female participants, an increasing self-concept was associated with better perceived performance in participants without a stereotype activation and with lower perceived performance in those with the activation. A post-hoc regression analysis was performed to analyze a possible interaction of self-concept and stereotype activation on females’ perceived mental rotation performance (Process macro Model 1 by Hayes, 2018). The interaction of stereotype activation and self-concept was not significant (*b* = −0.792, SE = 0.458, *p* = 0.089). For male adolescents, an increasing self-concept was associated with better perceived performance in adolescents with stereotype activation and with lower perceived performance in those without the activation. A post-hoc regression analysis (Process macro Model 1 by Hayes, 2018) revealed a significant interaction of stereotype activation and self-concept (*b* = 1.270, SE = 0.490, *p* = 0.012).

**Table 3:** Perceived mental rotation score predicted by gender (0 = male, 1 = female), stereotype activation (0 = without activation, 1 = with activation), and self-concept.

	*b*	SE	*t*	*p*	LLCI	ULCI
Constant	5.327	1.579	3.374	0.001	2.200	8.454
Self-concept	−0.280	0.441	−0.635	0.526	−1.155	0.593
Stereotype activation	−4.421	1.823	−2.424	0.016	−8.033	−0.809
Self-concept × SA	1.270	0.512	2.480	0.014	0.256	2.284
Gender	−3.714	1.896	−1.958	0.052	−7.470	0.042
Self-concept × Gender	0.888	0.539	1.646	0.102	−0.180	1.957
SA × Gender	7.203	2.375	3.032	0.003	2.498	11.909
Self-concept × Gender × SA	−2.062	0.676	−3.046	0.002	−3.403	−0.721

SA, stereotype activation; LLCI, lower-level confidence interval; ULCI, upper-level confidence interval. Confidence intervals are 95%.

## 4. Discussion

The present study analyzed the effects of gender stereotype activation on male and female adolescents’ actual and perceived mental rotation performance in consideration of their self-concept. Results showed that self-concept and stereotype activation affected male and female adolescents’ actual and perceived performance in distinct ways. For male adolescents with activated stereotypes, an increasing self-concept was associated with better performance. In male adolescents without the activation, performance was correlated negatively with self-concept. This interaction was only marginally significant. For females with stereotype activation, an increasing self-concept was associated with worse performance. For females without stereotype activation, it was associated with better performance.

Parallel results were found for the effects on adolescents’ perceived mental rotation performance. For males, a better self-concept was associated with higher perceived performance in adolescents with stereotype activation and with lower perceived performance without the activation. In contrast, a better self-concept was associated with higher perceived performance in female participants without stereotype activation and with lower perceived performance in those with the activation. The interaction of self-concept and stereotype activation was not significant for females.

### 4.1. Mental rotation performance

Regarding the influence of stereotype activation on mental rotation performance, our results did not support the hypothesis that females with a lower self-concept are more susceptible to stereotype threat activation. On the contrary: in female participants with stereotype activation, a lower self-concept was associated with better mental rotation performance. One explanation could be that female adolescents were already threatened by the material, which is perceived as rather male-stereotyped in elementary school children (Ruthsatz et al., 2017). This assumption is in line with a study with adults showing that spatial anxiety partially mediated the link between the sex of the participant and mental rotation performance (Alvarez-Vargas et al., 2020) and with Wang et al.’s (2018) study showing interactions of activated stereotypes and self-esteem. Additionally, the ability to rotate objects in one’s mind is male-stereotyped, too (Halpern et al., 2011). The manipulation check illustrated that females’ stereotype beliefs did not differ between participants with and without activation. Furthermore, spatial abilities could be of less importance for female adolescents. A stereotype threat could then have no effect.

For male adolescents, the stereotype activation had different effects depending on their self-concept. In males with stereotype activation, a higher self-concept was associated with better mental rotation performance. In contrast, in males without activation, a lower self-concept was associated with better performance. This interaction was only marginally significant. The result could help explain meta-analysis results (Doyle and Voyer, 2016) showing that stereotype lifts to males had no significant effect. One explanation for the interaction of self-concept and stereotype activation for male participants could be the congruence of their perception of their abilities and the experiment’s instruction. Males with a higher self-concept should be convinced of their abilities. When they get the information that their gender outperforms the opposite gender, it could motivate them. For example, it could support their competence needs or confirm their self-efficacy beliefs regarding their (spatial) ability. Accordingly, considering motivation could be an important next stepstone to better understand mental rotation performance. Failure could also mean that males’ self-worth is threatened (Walton and Cohen, 2003) so they could be encouraged and put more effort into the task. Males with a low self-concept could be in fear of failing in the task, while all other males would succeed. According to expectancy-value theories, this fear can then impair performance (e.g., Eccles and Wigfield, 2002). Another explanation could be that male adolescents with a lower self-concept could perceive themselves as less gender-typical. If they get the information that masculine individuals outperform feminine, they could assume that they will fail.

### 4.2. Perceived mental rotation performance

Very similar results were found for the perceived performance. The interaction of stereotype activation and self-concept had no significant effect on female adolescents’ perceived performance. There are different possible explanations for this result: females do not attribute any importance to spatial abilities and could perceive themselves as poor performers regardless of the additional information that their gender performs worse than the opposite gender.

On the one hand, males perceived their performance better after the stereotype activation when they had a higher self-concept. Males with a higher self-concept seem to rate their performance better because they got the information that their gender usually outperformed the opposite gender. This information could have strengthened their assessment. On the other hand, males with a lower self-concept perceived their performance worse when they got the activation. When male adolescents get the information that they should perform well, but they have a lower self-concept, they could be intimidated by the pressure. In their above-mentioned study, Martinot and Désert (2007) activated the gender identity of half of their adolescent participants and investigated the influence of gender stereotype beliefs on their math performance. No significant interaction of activation and stereotype beliefs appeared for girls. Boys who believed that girls outperformed boys in math rated their own performance negatively only when their gender identity was salient. This is partly in line with our results.

Future studies could investigate whether a stereotype lift activation for female adolescents could enhance their actual or perceived performance in spatial abilities (Moè and Pazzaglia, 2006). Moderating factors like self-concept or motivation should be analyzed as well. For male adolescents, these results could be used in the school context: when they have a high self-concept, gender stereotypes in masculine abilities could be emphasized to enhance their actual and perceived performance. Male adolescents with a lower self-concept should not focus on gender differences in stereotyped abilities. In general, it is possible to improve mental rotation abilities with training: Moè (2021) showed that short strategic or motivational training increased the mental rotation ability of older adolescents. Especially training girls and young women to believe that they can succeed in masculine stereotyped abilities when they put effort into it and use better strategies (Moè, 2016) can also be a promising approach to improve performance.

Overall, it can be assumed that boys and young men include spatial abilities in their self-concept, while this might not be as relevant for girls and young women. Female participants might refer to their language, arts, or music abilities in school to evaluate their self-concept (Valls, 2022). In general, a meta-analysis found lower correlations between self-concept and achievement for female than for male students (Möller et al., 2020).

### 4.3. Stereotype beliefs of mental rotation

Male adolescents with a higher self-concept reported stronger beliefs that mental rotation is male-stereotyped. This is in line with a study investigating the self-concept of black seventh- and eighth-graders (Evans et al., 2011): next to achievement, only males’ self-concept in math/science was predicted by their ratings of male stereotypes. Similarly, Igbo et al. (2015) report a stronger influence of gender stereotypes on students’ self-concepts for male than for female adolescents. Contrary to our results, Ertl et al. (2017) analyzed the STEM-specific self-concept and gender stereotypes in female STEM students and found a significant negative correlation. This difference could be explained by our sample being younger and not STEM-specific.

Correlations between stereotype beliefs and perceived performance were non-significant but negative for female and positive for male adolescents, especially in stereotype-activated male youths. Stronger beliefs that mental rotation is male-stereotyped were associated with higher perceived abilities in male adolescents. This illustrates that male participants either rate their performance according to their gender stereotypes or vice versa. Their stereotype beliefs could be influenced by their own perceived abilities. The result is in accord with a study investigating the influence of adolescents’ stereotype beliefs that girls are better in math on their perceived math performance (Martinot and Désert, 2007), where these stereotype beliefs had a non-significant negative effect on females and a significant negative effect on males’ perceived performance.

Furthermore, it is noteworthy that especially in male participants who were stereotypically activated, a better actual mental rotation performance was associated with a stronger belief that mental rotation is masculine stereotyped. On the one hand, it can be assumed that male adolescents who perform well in spatial tasks think that this is true for all males and, therefore, they develop stronger stereotype beliefs. On the other hand, a stronger stereotype belief could lead to better performance because they would then be more confident about their own abilities.

### 4.4. Limitations and future research

The study is limited by the fact that the questionnaires about stereotype beliefs and perceived abilities could not be analyzed for all masculine and feminine abilities and that they were filled out only once (after the mental rotation test was solved). Future studies should reverse this order and analyze the possible effects of stereotype beliefs and self-assessed abilities on adolescents’ actual mental rotation performance. Furthermore, we assessed adolescents’ self-concept in general, in school, and compared to others. Future studies could investigate their self-concept regarding spatial abilities. The data used in the present study were collected in a single country. Gender stereotypes and beliefs vary in different countries, which can affect performance in gendered abilities (Moè et al., 2021). Moreover, although a power analysis confirmed sufficient statistical power of our analyses, studies with larger sample sizes could confirm different correlations in the subgroups. Results should be interpreted cautiously because—as shown in the literature review—effects are dependent on the type of stereotype activation, mental rotation task, and participants’ age and cannot always be replicated.

### 4.5. Conclusion

To conclude, our results show that male adolescents with a high self-concept perform better in spatial tasks when they get the information that males are better. Males with a low self-concept perform worse with the stereotype activation than without it. Comparable results were found for males’ perceived spatial abilities. For females, self-concept was positively associated with performance in participants without the stereotype activation and negatively in adolescents who received the activation. No self-concept and stereotype activation effects appeared for female adolescents’ perceived spatial abilities.

## Data availability statement

The datasets presented in this study can be found in online repositories. The names of the repository/repositories and accession number(s) can be found at: https://osf.io/v9mqg/files/osfstorage/63ef456da3fade07eee7d3a1.

## Ethics statement

Ethical review and approval was not required for the study on human participants in accordance with the local legislation and institutional requirements. Written informed consent to participate in this study was provided by the participants’ legal guardian/next of kin.

## Author contributions

All authors contributed to the study’s conception and design. Material preparation and data collection were performed by MR, and analysis was conducted by MR and LS. The first draft of the manuscript was written by MR and all authors commented on previous versions of the manuscript. All authors contributed to the article and approved the submitted version.

## Conflict of interest

The authors declare that the research was conducted in the absence of any commercial or financial relationships that could be construed as a potential conflict of interest.

## Publisher’s note

All claims expressed in this article are solely those of the authors and do not necessarily represent those of their affiliated organizations, or those of the publisher, the editors and the reviewers. Any product that may be evaluated in this article, or claim that may be made by its manufacturer, is not guaranteed or endorsed by the publisher.

## References

[ref1] Alvarez-Vargas D. Abad C. Pruden S. M. (2020). Spatial anxiety mediates the sex difference in adult mental rotation test performance. Cogn. Res. 5, 31–17. doi: 10.1186/s41235-020-00231-8, PMID: 32712746 PMC7382671

[ref2] Bar-Haim Y. Lamy D. Pergamin L. Bakermans-Kranenburg M. J. van Ijzendoorn M. H. (2007). Threat-related attentional bias in anxious and nonanxious individuals: a meta-analytic study. Psychol. Bull. 133, 1–24. doi: 10.1037/0033-2909.133.1.1, PMID: 17201568

[ref3] Bauer R. Jost L. Jansen P. (2021). The effect of mindfulness and stereotype threat in mental rotation: a pupillometry study. J. Cogn. Psychol. 33, 861–876. doi: 10.1080/20445911.2021.1967366

[ref4] Cohen J. (1988). Statistical power analysis for the behavioural sciences. 2nd Edn. Hillsdale NJ: Academic Press.

[ref5] Cooke-Simpson A. Voyer D. (2007). Confidence and gender differences on the mental rotations test. Learn. Individ. Differ. 17, 181–186. doi: 10.1016/j.lindif.2007.03.009

[ref6] Delgado A. R. Prieto G. (2008). Stereotype threat as validity threat: the anxiety–sex–threat interaction. Intelligence 36, 635–640. doi: 10.1016/j.intell.2008.01.008

[ref7] Doyle R. A. Voyer D. (2016). Stereotype manipulation effects on math and spatial test performance: a meta-analysis. Learn. Individ. Differ. 47, 103–116. doi: 10.1016/j.lindif.2015.12.018

[ref8] Dunst B. Benedek M. Bergner S. Athenstaedt U. Neubauer A. C. (2013). Sex differences in neural efficiency: are they due to the stereotype threat effect? Pers. Individ. Differ. 55, 744–749. doi: 10.1016/j.paid.2013.06.007, PMID: 24092950 PMC3759843

[ref9] Eccles J. S. Wigfield A. (2002). Motivational beliefs, values, and goals. Annu. Rev. Psychol. 53, 109–132. doi: 10.1146/annurev.psych.53.100901.135153 11752481

[ref10] Ertl B. Luttenberger S. Paechter M. (2017). The impact of gender stereotypes on the self-concept of female students in STEM subjects with an under-representation of females. Front. Psychol. 8:703. doi: 10.3389/fpsyg.2017.00703, PMID: 28567022 PMC5434750

[ref11] Estes Z. Felker S. (2012). Confidence mediates the sex difference in mental rotation performance. Arch. Sex. Behav. 41, 557–570. doi: 10.1007/s10508-011-9875-5, PMID: 22130691

[ref12] Evans A. B. Copping K. E. Rowley S. J. Kurtz-Costes B. (2011). Academic self-concept in black adolescents: do race and gender stereotypes matter? Self Ident. 10, 263–277. doi: 10.1080/15298868.2010.485358, PMID: 21552362 PMC3086770

[ref13] Faul F. Erdfelder E. Lang A.-G. Buchner A. (2007). G*power 3: a flexible statistical power analysis program for the social, behavioral, and biomedical sciences. Behav. Res. Methods 39, 175–191. doi: 10.3758/BF03193146, PMID: 17695343

[ref14] Field A. (2013). Discovering statistics using IBM SPSS statistics. Los Angeles: SAGE.

[ref15] Flore P. C. Wicherts J. M. (2015). Does stereotype threat influence performance of girls in stereotyped domains? A meta-analysis. J. School psych. 53, 25–44. doi: 10.1016/j.jsp.2014.10.002 25636259

[ref16] Gerstenberg F. X. Imhoff R. Schmitt M. (2012). ‘Women are bad at math, but I'M not, am I?’ Fragile mathematical self–concept predicts vulnerability to a stereotype threat effect on mathematical performance. Europ J Pers 26, 588–599. doi: 10.1002/per.

[ref18] Good C. Aronson J. Inzlicht M. (2003). Improving adolescents’ standardized test performance: an intervention to reduce the effects of stereotype threat. J. Appl. Develop Psychol. 24, 645–662. doi: 10.1016/j.appdev.2003.09.002

[ref19] Guizzo F. Moè A. Cadinu M. Bertolli C. (2019). The role of implicit gender spatial stereotyping in mental rotation performance. Acta Psychol. 194, 63–68. doi: 10.1016/j.actpsy.2019.01.013, PMID: 30753946

[ref20] Halpern D. F. Straight C. A. Stephenson C. L. (2011). Beliefs about cognitive gender differences: accurate for direction, underestimated for size. Sex Roles 64, 336–347. doi: 10.1007/s11199-010-9891-2

[ref21] Hausmann M. (2014). Arts versus science—academic background implicitly activates gender stereotypes on cognitive abilities with threat raising men’s (but lowering women’s) performance. Intelligence 46, 235–245. doi: 10.1016/j.intell.2014.07.004

[ref22] Hausmann M. Schoofs D. Rosenthal H. E. Jordan K. (2009). Interactive effects of sex hormones and gender stereotypes on cognitive sex differences—a psychobiosocial approach. Psychoneuroendocrino 34, 389–401. doi: 10.1016/j.psyneuen.2008.09.019, PMID: 18992993

[ref23] Hayes A. F. (2018). Introduction to mediation, moderation, and conditional process analysis, second edition (methodology in the social sciences). 2nd Edn. New York: Guilford Press.

[ref24] Heil M. Jansen P. Quaiser-Pohl C. Neuburger S. (2012). Gender-specific effects of artificially induced gender beliefs in mental rotation. Learn. Individ. Differ. 22, 350–353. doi: 10.1016/j.lindif.2012.01.004

[ref27] Igbo J. N. Onu V. C. Obiyo N. O. (2015). Impact of gender stereotype on secondary school students’ self-concept and academic achievement. SAGE Open 5:215824401557393. doi: 10.1177/2158244015573934

[ref28] Lauer J. E. Yhang E. Lourenco S. F. (2019). The development of gender differences in spatial reasoning: a meta-analytic review. Psychol. Bull. 145, 537–565. doi: 10.1037/bul0000191, PMID: 30973235

[ref29] Lovakov A. Agadullina E. R. (2021). Empirically derived guidelines for effect size interpretation in social psychology. Eur. J. Soc. Psychol. 51, 485–504. doi: 10.1002/ejsp.2752

[ref30] Martinot D. Désert M. (2007). Awareness of a gender stereotype, personal beliefs and self-perceptions regarding math ability: when boys do not surpass girls. Soc. Psychol. Educ. 10, 455–471. doi: 10.1007/s11218-007-9028-9

[ref31] Moè A. (2016). Teaching motivation and strategies to improve mental rotation abilities. Intelligence 59, 16–23. doi: 10.1016/j.intell.2016.10.004

[ref32] Moè A. (2018a). Mental rotation and mathematics: gender-stereotyped beliefs and relationships in primary school children. Learn. Individ. Differ. 61, 172–180. doi: 10.1016/j.lindif.2017.12.002

[ref33] Moè A. (2018b). Effects of group gender composition on mental rotation test performance in women. Arch. Sex. Behav. 47, 2299–2305. doi: 10.1007/s10508-018-1245-0, PMID: 29858725

[ref34] Moè A. (2021). Doubling mental rotation scores in high school students: effects of motivational and strategic trainings. Learn Instruc. 74:101461. doi: 10.1016/j.learninstruc.2021.101461

[ref35] Moè A. Hausmann M. Hirnsten M. (2021). Gender stereotypes and incremental beliefs in STEM and non-STEM students in three countries. Relationships with performance in cognitive tasks. Psychol. Res. 85, 554–567. doi: 10.1007/s00426-019-01285-0, PMID: 31960121

[ref36] Moè A. Pazzaglia F. (2006). Following the instructions!: effects of gender beliefs in mental rotation. Learn. Individ. Differ. 16, 369–377. doi: 10.1016/j.lindif.2007.01.002

[ref37] Möller J. Zitzmann S. Helm F. Machts N. Wolff F. (2020). A meta-analysis of relations between achievement and self-concept. Rev. Educ. Res. 90, 376–419. doi: 10.3102/0034654320919354

[ref38] Neuburger S. Jansen P. Heil M. Quaiser-Pohl C. (2015). A threat in the classroom. Z. Psychol. 220, 61–69. doi: 10.1027/2151-2604/a000097

[ref39] Peters M. Laeng B. Latham K. Jackson M. Zaiyouna R. Richardson C. (1995). A redrawn Vandenberg and Kuse mental rotations test-different versions and factors that affect performance. Brain Cogn. 28, 39–58. doi: 10.1006/brcg.1995.1032, PMID: 7546667

[ref40] Rahe M. Jansen P. (2022). Sex differences in mental rotation: the role of stereotyped material, perceived performance and extrinsic spatial ability. J. Cogn. Psychol. 34, 400–409. doi: 10.1080/20445911.2021.2011896

[ref41] Rahe M. Jansen P. (2023). Does mindfulness help to overcome stereotype threat in mental rotation in younger and older adolescents? Psychol. Res. 87, 624–635. doi: 10.1007/s00426-022-01666-y, PMID: 35302181 PMC9928811

[ref42] Rahe M. Quaiser-Pohl C. (2019). Mental-rotation performance in middle and high-school age: influence of stimulus material, gender stereotype beliefs, and perceived ability of gendered activities. J. Cogn. Psychol. 31, 594–604. doi: 10.1080/20445911.2019.1649265

[ref43] Rahe M. Quaiser-Pohl C. (2023). Can (perceived) mental-rotation performance mediate gender differences in math anxiety in adolescents and young adults? Math. Educ. Res. J. 35, 255–279. doi: 10.1007/s13394-021-00387-6

[ref44] Rahe M. Ruthsatz V. Quaiser-Pohl C. (2021). Influence of the stimulus material on gender differences in a mental-rotation test. Psychol. Res. 85, 2892–2899. doi: 10.1007/s00426-020-01450-w, PMID: 33237389

[ref45] Rossi S. Xenidou-Dervou I. Simsek E. Artemenko C. Daroczy G. Nuerk H. C.. (2022). Mathematics-gender stereotype endorsement influences mathematics anxiety, self-concept, and performance differently in men and women. Ann. N. Y. Acad. Sci. 1513, 121–139. doi: 10.1111/nyas.14779 35429357 PMC9545177

[ref46] Ruthsatz V. Neuburger S. Rahe M. Jansen P. Quaiser-Pohl C. (2017). The gender effect in 3D-mental-rotation performance with familiar and gender-stereotyped objects—a study with elementary school children. J. Cogn. Psychol. 29, 717–730. doi: 10.1080/20445911.2017.1312689

[ref47] Schöne C. Dickhäuser O. Spinath B. Stiensmeier-Pelster J. (2002). Skalen zur Erfassung des schulischen Selbstkonzepts: SESSKO. Göttingen: Hogrefe.

[ref48] Shepard R. N. Metzler J. (1971). Mental rotation of three-dimensional objects. Science 171, 701–703. doi: 10.1126/science.171.3972.701, PMID: 5540314

[ref49] Steele C. M. Aronson J. (1995). Stereotype threat and the intellectual test performance of African Americans. J. Pers. Soc. Psychol. 69, 797–811. doi: 10.1037/0022-3514.69.5.797 7473032

[ref50] Tomasetto C. Alparone F. R. Cadinu M. (2011). Girls’ math performance under stereotype threat: the moderating role of mothers’ gender stereotypes. Dev. Psychol. 47, 943–949. doi: 10.1037/a0024047, PMID: 21744956

[ref51] Valls M. (2022). Gender differences in social comparison processes and self-concept among students. Front. Educ. 6:815619. doi: 10.3389/feduc.2021.815619

[ref52] Vandenberg S. G. Kuse A. R. (1978). Mental rotations, a group test of three-dimensional spatial visualization. Percept. Mot. Skills 47, 599–604. doi: 10.2466/pms.1978.47.2.599, PMID: 724398

[ref53] Voyer D. Voyer S. Bryden M. P. (1995). Magnitude of sex differences in spatial abilities: a meta-analysis and consideration of critical variables. Psychol. Bull. 117, 250–270. doi: 10.1037/0033-2909.117.2.250, PMID: 7724690

[ref54] Wai J. Lubinski D. Benbow C. P. (2009). Spatial ability for STEM domains: aligning over 50 years of cumulative psychological knowledge solidifies its importance. J. Educ. Psychol. 101, 817–835. doi: 10.1037/a0016127

[ref55] Walton G. M. Cohen G. L. (2003). Stereotype lift. J. Exp. Soc. Psychol. 39, 456–467. doi: 10.1016/S0022-1031(03)00019-2

[ref56] Wang Q. Yu G. Pedram C. Chen C. (2018). Higher self-esteem is linked to greater stereotype threat among academically low-achieving students. Soc. Behav. Personal. 46, 1123–1132. doi: 10.2224/sbp.5410

