# Supplementary material for: Self-concept explains gender differences in mental rotation performance after stereotype activation
Source: Front Psychol. 2023 May 15;14:1168267. doi: 10.3389/fpsyg.2023.1168267 (PMC10226650; doi:10.3389/fpsyg.2023.1168267)
Supplement: Supplementary file 1 [file Table_1.pdf]

Supplements

Table A

Descriptive statistics of the study variables for male and female adolescents.

	Males		Females	
	M	*SD*	*M*	*SD*
MR	4.56	3.67	3.06	2.16
Self-concept	3.48	0.66	3.38	0.64
Perceived ability of MR	4.32	1.20	3.71	1.18
Stereotype belief of MR	0.75	1.15	0.69	1.19

Note. MR = mental rotation performance.
